# 

**Published:** 1899-01-21

**Authors:** 


**Notices of Births, Deaths, and Marriages** are inserted in this column at a charge of 2s. 6d. for an announcement not exceeding four lines.

### NOTICE TO CORRESPONDENTS.

All MS., Letters, Books for Review, and other matters intended for the Editor should be addressed The Editor, "The Hospital," The Hospital Building, 28 & 29, Southampton Street, Strand, W.C.
The Editor cannot undertake to return rejected MS., even when accompanied by stamped directed envelope.

**Notice to Advertisers and Subscribers.**

All Advertisements, Orders for Copies of The Hospital, and Business Communications should be addressed to **THE MANAGER (Not to the Editor),** The Hospital, The Hospital Building, 28 & 29, Southampton Street, Strand, London, W.C.

The Cost of Subscription, post free, is as follows:—Per week, 2½d.; per quarter, 2s. 8d.; per half-year, 5s. 3d.; per annum, 10s. 6d. Foreign:—Per annum, 15s. 2d., payable, in all cases, in advance.

**NOTICE.—Vol. XXIV. of "The Hospital" (April, 1898, to September, 1898), in handsome cloth binding, is now on sale at the Office of this Journal, price 7s. 6d. Cases for binding the Numbers contained in this Volume 1s. 6d. Index to Vol. XXIV., 2½d., post free.**

**The Monthly Part for February will be ready on February 1st. Price 6d., by post 8d.**

### BOOKS RECEIVED.

Chatto and Windus.

"Herbert Fry's Royal Guide to the London Charities." Edited by John Lane.

The Scientific Press.

"Photo-Micrography." By Edmund J. Spitta, L.R.C.P., M.R.C.S., F.R.A.S.

SINCE the crusade of the National Association for the Prevention of Tuberculosis has commenced several Boards of Guardians have discussed the question of taking action, and it is probable that a conference of the Metropolitan Boards will take place relative to the establishment of joint sanitoria.

---

ALDERMAN TRELOAR writes that the subscriptions already received by him have been sufficient to pay for the banquet at the Guildhall, for 4,020 hampers to be sent to poor crippled children in the metropolis, and to leave a balance of about £25. He is appealed to for 300 more hampers, and will send them out this week. This will leave a deficiency of about £35. He states that 300 poor little ones have been led to believe that hampers would be sent to them, and asks that the deficiency may be made good, so that next year an endeavour to send out 5,000 hampers may be made.

### SCRAPS AND GLEANINGS.

ONE hundred and twenty-eight pounds was collected this year in the Hull Sunday schools in aid of the Hospital Sunday Fund.

THE Norwich Hospital Sunday and Saturday Fund has this year collected £1,500 for distribution amongst ten medical charities.

HER Majesty the Queen has commanded that 100 lb. of cast linen be forwarded for the service of the Cancer Hospital, Brompton.

OWING to the increasing work devolving on the Victoria Infirmary, Glasgow, it has been found necessary to extend the nurses' quarters. This extension is to be begun in February.

LADY GRAY, the widow of Sir William Gray, has handed over to the Hartlepools Hospital the sum of £700 for the purpose of carrying out the proposed extensions of the nurses' quarters.

THE Peterborough Infirmary is to have £320 this year from the Hospital Saturday Fund, making the amount granted to the infirmary by the Saturday Fund during the last 14 years, £4,100.

THE annual church collections in aid of the Aberdeen Royal Infirmary this year show that the returns from 60 churches and meeting-places amount to £1 119 11s., which is an increase of £182 odd over last year.

THE Committee of the Children's Cottage Hospital, Coldash, have added the following to their rules: "Children who have not been, and whose parents are unwilling that they should be vaccinated, cannot be admitted."

THE number of cases treated in the wards of the Victoria Infirmary, Glasgow, last year was 1,720, being the largest number in any one year since the infirmary was opened. An increase has also taken place in all the branches.

IT is stated that there is to be a meeting of subscribers to the Devon and Exeter Hospital to receive a report from the special committee appointed in November, 1897, to recommend methods by which the expenditure could be reduced.

THE difficulty at the Swansea Hospital, which resulted in the resignation of one of the ophthalmic surgeons, has been smoothed over; the out-patients, officers, &c., have gone back to their old quarters, and Dr. Davidson, the surgeon in question, has withdrawn his resignation.

THE new building for St. Mary's Hospital, Manchester, has been commenced. The accommodation in the present hospital, both for nurses and patients, is now taxed to its utmost, and a new home in a house adjoining the hospital has been taken for the nurses, at an increased expense of £30 a year.

THE total cost of the improvements and alterations at Radcliffe Infirmary, Oxford, which are now approaching completion, will not, it is estimated, fall far short of £5,500. The committee have had to entrench on their capital to meet this expenditure, and they now appeal for increased annual subscriptions to meet the deficiency in income from investments thus created.

AT a meeting held for the purpose of considering the proposal to erect a convalescent hospital for scarlet fever patients in connection with the City and County Infirmary, Perth, it was resolved to recommend to the various local authorities that an arrangement should be come to for the acquisition of ground at Hillend, and the building thereon of the convalescent hospital.

THERE does not seem to be very general satisfaction at the proposal of the Southampton Guardians to increase the accommodation provided in their workhouse so as to provide 300 beds for the sick. It is considered that such accommodation will be far in excess of requirements, and that the Guardians should not accede to the demand made by the Local Government Board that a new infirmary should be built.

WE understand that by the award of a diploma and gold medal at the South London Food and Drink (Temperance) Exhibition just concluded, Fromms Extract Company (Limited) have now gained no less than 26 gold medals and prizes, many of them at the leading exhibitions of the world, a fact which goes far to prove the assertion of the firm, that their vegetable extract and vegetable soup tablets are rapidly attaining a leading position amongst the foods of the people.

AS a result of the action of the authorities of the Macclesfield Infirmary, the Health Committee of the Corporation have passed a resolution that in future all cases of infectious diseases arising amongst patients in the Macclesfield Infirmary who are *bona fide* inhabitants of the borough be admitted to the Infectious Diseases Hospital of the Corporation if such hospital be available, free of charge to the infirmary. The committee are much to be congratulated on this result of their action.

IN regard to the management of the Devon and Exeter Hospital and the great increase of expenditure over income, the Executive Committee of the Hospital Saturday Fund have forwarded a communication to the authorities suggesting that a meeting of the Governors should be called to appoint a Special Committee, who should recommend what administrative alterations were necessary. So far, however, the Hospital Committee do not appear to have done anything in this direction.

THROUGH the instrumentality of the County Council, a conference has been held at Lewes of representatives throughout East Sussex to consider the question of the provision of sufficient isolation hospitals. A resolution was passed that it was expedient that the sanitary authorities of the county should be invited to co-operate in providing, separately or in combination between two or more of them for the use of all districts not already so provided, hospitals suitably placed for the treatment of infectious diseases other than small-pox.

THE British Vice-Consul at Los Angeles, California, is appealing to English friends for funds in support of the Children's Home Society of Los Angeles. This gentleman (Mr. White Mortimer) is president of this society, and in a circular letter he has sent out he gives some interesting particulars of the charity, the object of which is to take charge of orphaned or abandoned children under eight years of age, and maintain them all until responsible persons can be found to adopt them. Many of the children so taken charge of are merely infants, children of English parents, and it costs £10 on an average to save each child, and find it a good home.

It is proposed to perpetuate the memory of the late Dr. Brett, of Bridlington, by endowing a cot in the Lloyd Cottage Hospital, for which a sum of £300 will be required.

It may not be generally known that several of the shipbuilding firms in and around Sunderland have for some time given a special donation to the Sunderland Infirmary based on a certain percentage of the wages paid. It is suggested that this plan should be generally adopted, which, on a basis of 1s. per cent. on £800,000, the amount computed to be paid annually by the Sunderland and district engineers and shipbuilders, would result in a subscription of £400 per annum to the infirmary. Such an addition to the funds would be very welcome, as the Sunderland Infirmary is labouring under a debt of £1,350, which has already been reduced by £250 by the handsome donation from Mr. Short.

It is stated that the contractors for the extension of the Bulawayo Hospital have stopped work owing to lack of funds. It has been decided that the sureties should be called upon to complete the work.

The doll show that was recently held at Langham, Norfolk, with the object of helping towards the renovation of the parish room and providing dolls for all the girls in the Norfolk hospitals and convalescent homes, has proved a far greater success than was anticipated. The entrance money was devoted to the renovation of the parish room, which was crowded with visitors throughout the show. There were 205 dolls sent in, but as only 93 were required it was resolved to sell some of them, and buy toys for the boys in the Norfolk institutions, the remainder of the dolls being sent to the children's hospitals at Brighton and Chelsea, whence several of the exhibits had come.

### THE STUDENT OF MEDICINE.

### APPOINTMENTS.

A. G. L. CAMPBELL, appointed Medical Officer for the Eighth District of the Newmarket Union. P. S. Campkin, L.D.S.Edin., appointed Dental House Surgeon at Guy's Hospital. D. Davies-Jones, M.B., M.S.Edin., appointed Surgeon to the Lower Duffryn Colliery, Mountain Ash. Sarah Gray, L.R.C.P. & S Edin., L.F.P.S.Glasg., appointed Assistant Surgeon to the Nottingham Hospital for Women. J. Hodges, M.R.C.S.Eng., appointed Medical Officer for the Edmonton District of the Edmonton Union. J. E. Knox, M.B., C.M.Edin., appointed Medical Officer for the East and West Molesey Urban District. E. N. Mason, L.D.S.Eng., appointed Dental House Surgeon at Guy's Hospital. F. Pershouse, M.R.C.S., L.R.C.P., appointed Medical Officer for the Bradwell District of the Maldon Union. A. G. G. Plumley, M.B.Lond., M.R.C.S., L.R.C.P., L.D.S.Eng., appointed Dental House Surgeon at Guy's Hospital. F. L. Pochin, M.B.Edin., appointed Medical Officer for the Raynham District of the Walsingham Union. A. M. Rattray, M.B., C.M.Edin., appointed Assistant Medical Officer to the City of Newcastle Asylum, Gosforth. J. S. Ross, M.B., B.Ch.Vict., appointed House Surgeon and Resident Obstetric Assistant Surgeon at St. Mary's Hospital, Manchester. A. C. F. Smith, L.R.C.P., L.R.C.S.Edin., L.F.P.S.Glasg., appointed Medical Officer for the Northern District of the Devonport Union. C. C. Smith, M.B., appointed Medical Officer for the Redditch District of the Bromsgrove Union. H. C. Thomson, M.D.Lond., M.R.C.P., appointed Pathologist and Curator of the Museum to the Middlesex Hospital. W. K. Walls, M.B.Lond., M.R.C.S., appointed Obstetric Assistant Surgeon to St. Mary's Hospital, Manchester.

Crown 8vo., Paper Covers, 6d.

### THE OPEN-AIR TREATMENT OF PHTHISIS.

**A Visit to Falkenstein.**

By RICHARD GREENE, F.R.C.P.Ed.

London: THE SCIENTIFIC PRESS (LTD.), 28 & 29, Southampton Street, Strand, W.C.

